# Erectile function in men with end-stage liver disease improves after living donor liver transplantation

**DOI:** 10.1186/s12894-015-0078-6

**Published:** 2015-08-13

**Authors:** You-Chiuan Chien, Heng-Chieh Chiang, Ping-Yi Lin, Yao-Li Chen

**Affiliations:** Division of urology, Department of Surgery, Changhua Christian Hospital, No.135, Nansiao St. Changhua city, Changhua county, 50006 Taiwan; Transplant Medicine & Surgery Research Centre, Changhua Christian Hospital, Changhua, Taiwan; School of Medicine, Kaohsiung Medical University, Kaohsiung, Taiwan; Department of General Surgery, Changhua Christian Hospital, No.135 Nan-Hsiao Street, Changhua county, 50006 Taiwan

## Abstract

**Background:**

Impaired liver function in men can result in erectile dysfunction or hypogonadism or both. We investigated whether living donor liver transplantation (LDLT) results in improvement in male sexual function.

**Methods:**

A total of 58 patients with end-stage liver disease (ESLD) were included in this prospective, cross-sectional study. Erectile function was measured before and after LDLT using a five-item modified version of the International Index of Erectile Function scale (IIEF-5) and hypogonadism was evaluated before and after LDLT using the Androgen Deficiency in the Aging Male (ADAM) questionnaire. Differences in mean values from the questionnaires before and after the operation were than evaluated to determine whether there is an association between LDLT and improvement in sexual function.

**Results:**

We found that mean IIEF-5 scores significantly increased after LDLT (from 11.7 ± 7.7 before LDLT to 14.7 ± 7.5 after LDLT, *p* <0.01), indicating that the operation played a role in improving erectile function. In addition, the prevalence of hypogonadism among the patients with ESLD decreased markedly after liver transplantation (hypogonadism before LDLT, n = 41 versus hypogonadism after LDLT, n = 31, *p* = 0.03). Patients with hypogonadism reported a higher prevalence of erectile dysfunction after LDLT than patients without hypogonadism (*p* <0.01).

**Conclusions:**

LDLT results in improvement in erectile function. In addition, improvement in erectile function is associated with self-reported absence of hypogonadism.

## Background

Liver transplantation is considered standard curative therapy for patients with advanced hepatocellular carcinoma and for patients with alcohol-related end-stage liver disease (ESLD). Improvements in surgical techniques as well as medical therapy have resulted in better long-term survival outcomes for patients who require liver transplantation. However, patients with chronic disease tend to care more about quality of life than about the duration of life. Sexual function, a component of quality of life, is a major concern for men with ESLD and for those who have received liver transplantation [1, 2]. Damage to the hypothalamic-pituitary-gonadal axis and impaired liver function are the main causes of sexual dysfunction in patients with cirrhosis [1, 3]. Studies have shown that orthotopic liver transplantation (OLT) can result in restoration of the hypothalamic-pituitary-gonadal axis [4, 5].

Religious beliefs, cultural values, educational systems and differences in legislation associated with brain death help to explain why OLT is not as common in Asian countries as in Western countries [6]. Increased need for liver transplantation and a shortage of deceased donors led to the development of living donor liver transplantation (LDLT) as an alternative to OLT. Although long-term survival rates, organ rejection rates, and rates of hepatitis C virus (HCV) recurrence have been shown to be more or less equal between LDLT and OLT, studies have shown that LDLT is associated with a shorter ischemic time, a lower mortality rate, and greater peri-operative benefits than OLT [7].

The prevalence of erectile dysfunction is high in patients with ESLD [8]. Although the gold standard for determining the presence of hypogonadism in men is measuring free testosterone level, that test is not widely performed in clinical laboratories. An alternative to measuring free testosterone in serum is to administer the self-report Androgen Deficiency in the Aging Male (ADAM) questionnaire, which is designed to assess symptoms associated with androgen deficiency and, therefore, can be used as a tool to detect and measure the degree of hypogonadism. The ADAM questionnaire is a simple, non-invasive measure that has been shown to have a high sensitivity and a relatively acceptable specificity [9, 10].

Erectile dysfunction (ED) is defined as the inability to develop or maintain an erection during sexual intercourse and is the main domain of sexual dysfunction in men [8]. Although ED is an important quality-of-life issue, it is often underestimated by patients and physicians. The five-item International Index of Erection Function (IIEF-5) questionnaire provides a measurable scale with which to estimate the true prevalence of ED [11]. To the best of our knowledge, no studies have evaluated the effect of LDLT on sexual function in men. Thus, in this study, we measured the differences in pre- and post-surgical IIEF-5 and ADAM questionnaire scores to investigate whether LDLT affects sexual function in men.

## Methods

### Patients

**Fig. 1:**
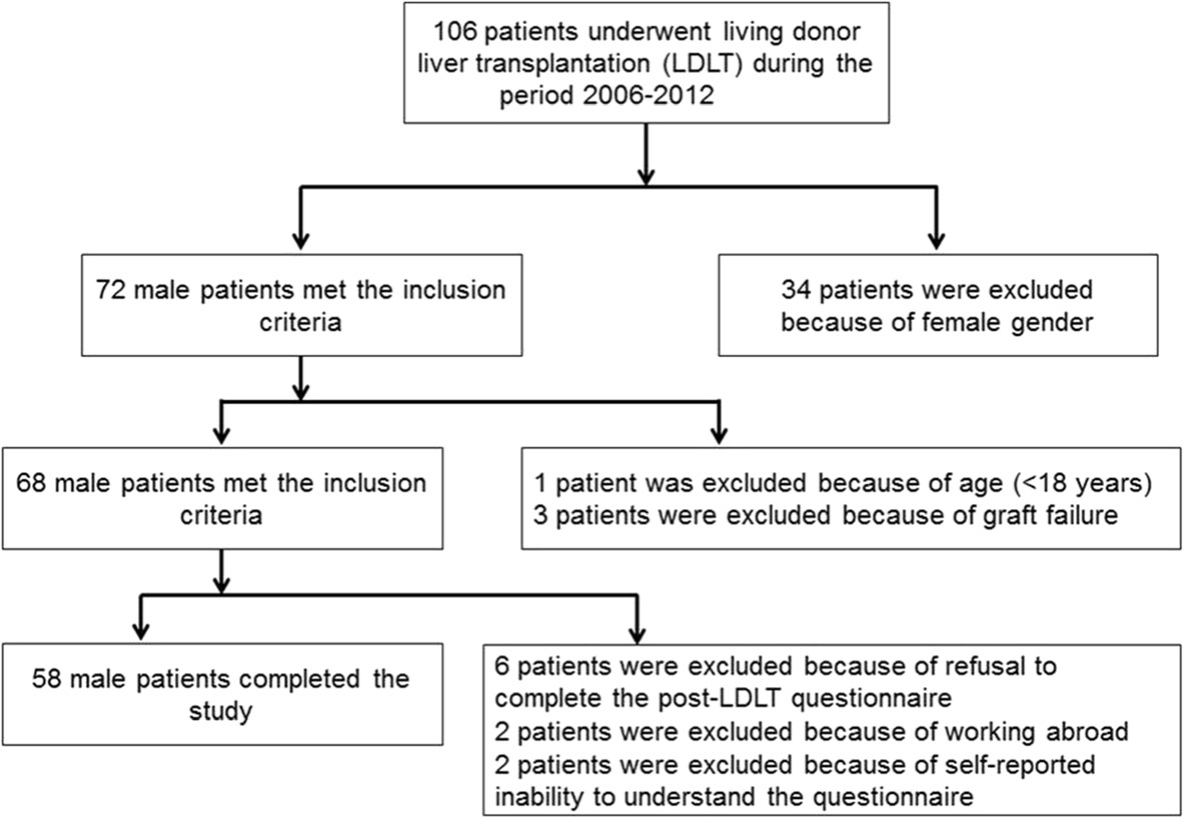
Scheme of patient selection in the study

The patients in this prospective cohort study comprised 68 men with end-stage liver disease who were scheduled to undergo LDLT at the Changhua Christian hospital (Changhua, Taiwan) during the period 2006–2012. Written informed consents were obtained from the patients. The five-item International Index of Erection Function (IIEF-5) questionnaire, designed to evaluate erectile dysfunction, and the Androgen Deficiency in the Aging Male (ADAM) questionnaire, designed to measure the degree of hypogonadism, were administered to the patients before LDLT and six months after the operation. Patients were required to complete the IIEF-5 questionnaire in the outpatient department about six to seven months after transplantation. At the end of the study period, 10 men were excluded from the study because of because of working abroad (n = 2), because they refused to complete the questionnaires (n = 6), or because of self-reported inability to understand the questionnaire during the post-operative period (n = 2). Therefore, data on 58 patients were evaluated (Fig. 1).

The Institutional Review Board of the Changhua Christian hospital approved the study.

### Inclusion and exclusion criteria

Adult (≥18 years) male recipients of living donor liver transplant who had completed a 6-month follow-up assessment were considered eligible for inclusion. Exclusion criteria included female gender, age < 18 years, liver graft failure, refusal to complete the post-LDLT questionnaires, and loss to follow-up for any reason.

### Survey

An introductory letter comprising a detailed description of the methodology of the study, the future consequences of the study, the name of the contact person of the study, and associated information was presented to each participant before LDLT. Data collection was completed by two doctors in the urology division of the surgery department via telephone or face-to-face interview. Etiology of liver disease (hepatitis B virus, hepatitis C virus, or alcoholism), alcohol intake history, the presence of diabetes and demographic data were obtained through chart review.

### Main outcome measures

#### Measures

The International Index of Erectile Function (IIEF), also known as the Sexual Health Inventory for Men (SHIM), is a widely used questionnaire for the evaluation of male sexual function and is recommended as a primary endpoint for diagnostic evaluation of ED severity by the National Institutes of Health Consensus Panel on Impotence [12]. The IIEF-5 is a modified version of the IIEF comprising five items instead of 15 items and was designed to shorten the time needed to complete the survey. The IIEF-5 questionnaire shows the presence and severity of ED over the past six months [13]. Studies have shown that the validity and sensitivity of the IIEF-5 are similar to those of the original IIEF scale [11, 14]. The IIEF-5 is graded on a scale from 1 to 5 points for each of the five items. The total score therefore ranges from 5 to 25. The primary score is classified into five categories of severity, namely severe (score 5–7), moderate (score 8–11), mild-to-moderate (score 12–16), mild (score 17–21), and no ED (score 22–25). The Androgen Deficiency in the Aging Male (ADAM) questionnaire is a self-report, ten-item scale designed to evaluate the degree of androgen deficiency [10]. ADAM consists of 10 questions and answering “yes” to question 1 or 7 or to 3 or more of the questions is regard as an indication of possible hypogonadal status. ADAM has been shown to be a highly sensitive but poorly specific measure for determining androgen deficiency [15]. Nonetheless, Tancredi et al. found that hormone analysis of blood samples correlated with ADAM scores [9]. Patients who reported decreased libido or inadequate erectile “strength” and patients who provided positive answers to 3 of the other questions listed in the questionnaire were considered to have androgen deficiency. Both questionnaires were given to patients before LDLT and at least six months after the operation to evaluate whether LDLT had an effect on erectile dysfunction and hypogonadism.

#### Statistical analysis

Categorical data were compared by the McNemar's test or the Chi-square test. Differences in continuous variables were compared by the Wilcoxon signed-rank test. A *p* value of less than 0.05 was considered to indicate statistical significance. All statistical analyses were performed on a personal computer with the statistical package SPSS for Windows (Version 18.0, SPSS, Chicago, Il).

## Results

During the period November 2012 to May 2013, 58 of the 68 (85 %) adult men who were eligible to participate in the study completed both questionnaires before and after LDLT. Reasons for not completing the questionnaires during the post-operative period included refusal to participate (n = 6), working abroad (n = 2), and self-reported inability to understand the questionnaire (n = 2). Table 1 summarizes the general characteristics of the patients who participated in the study. The mean age at the time of LDLT was 53.86 ± 7.53 years. Alcohol abuse was self-reported in 17 patients before LDLT and in 4 after the operation. In addition, 15 of the patients had diabetes mellitus before surgery and 27 had the disorder after LDLT (Table 1).

**Table 1:** Characteristics of the population before and after transplantation

	Before transplantation N (%)	After transplantation N (%)	*p* value
Age (years; mean ± SD)	53.86 ± 7.53	55.02 ± 7.33	< 0.001
Alcohol abuse	17 (29.3)	4 (6.9)	< 0.001
HBV	34 (58.6)	1 (1.7)	< 0.001
HCV	17 (29.3)	6 (10.3)	0.001
Diabetes	15 (25.9)	27 (46.6)	< 0.001
IIEF5 score (mean ± SD)	11.7 ± 7.7	14.7 ± 7.5	0.001
Suspected hypogonadism via ADAM questionnaire	41 (70.6)	31 (53.4)	0.031

### Change in erectile dysfunction

**Fig. 2:**
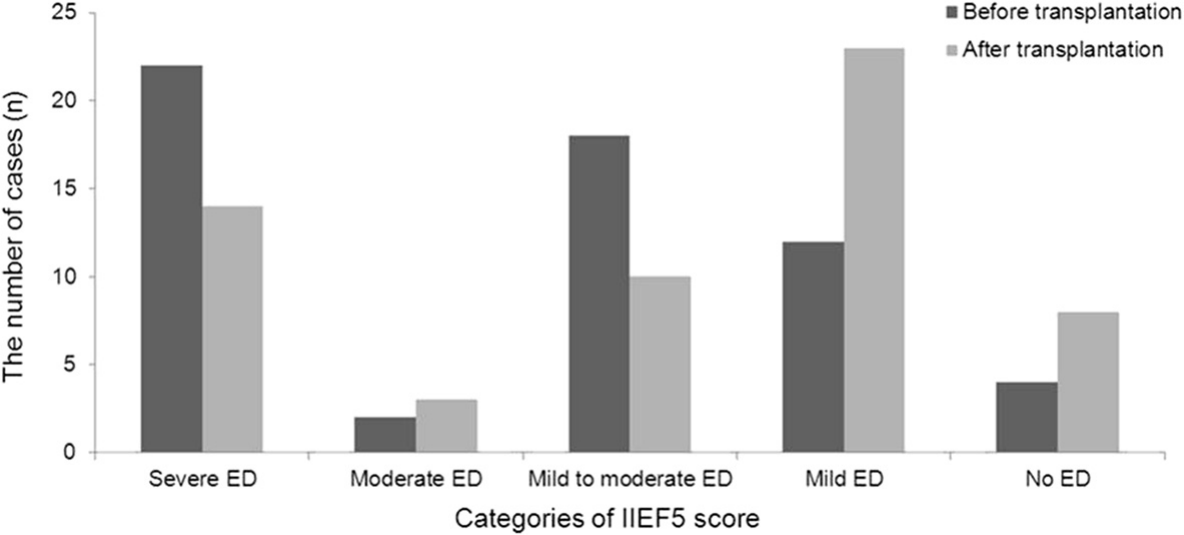
Prevalence of erectile dysfunction prior to and after liver transplantation

We found that mean IIEF-5 scores significantly increased after LDLT (from 11.7 ± 7.7 before LDLT to 14.7 ± 7.5 after LDLT, *p* <0.01), indicating that the operation played a role in improving erectile function. Overall, 28 patients reported improvement in erectile function, 23 patients reported no change in erectile function and 7 patients reported worse erectile function. We also found that the degrees of ED changed among the study population after surgery (Table 2). The percentage of patients with IIEF-5 scores indicative of severe ED or mild-to-moderate ED was markedly lower after LDLT (24.1 % vs. 37.9 % and 17.2 % vs. 31.0 % respectively) and the percentage of patients with IIEF-5 scores indicating no ED was noticibly higher after LDLT (13.8 % vs. 6.9 %). Interestingly, however, the percentage of patients with scores indicative of moderate ED or mild ED was higher after LDLT (5.2 % vs. 3.4 % and 39.7 % vs. 20.7 %, respectively) (Table 2) (Fig. 2).

**Table 2:** Changes of erectile dysfunction prior to and after liver transplantation

		Before transplantation N (%)	After transplantation N (%)	*p* value
Categories of IIEF5 score	Severe ED	22 (37.9)	14 (24.1)	< 0.001
	Moderate ED	2 (3.4)	3 (5.2)	
	Mild to moderate ED	18 (31.0)	10 (17.2)	
	Mild ED	12 (20.7)	23 (39.7)	
	No ED	4 (6.9)	8 (13.8)

### Change in hypogonadism

The prevalence of hypogonadism among the patients with ESLD decreased markedly after liver transplantation based on the scores from the ADAM questionnaires administered before and after surgery (hypogonadism before LDLT, n = 41 vs. hypogonadism after LDLT, n = 31, *p* = 0.031).

### Association of erectile dysfunction and hypogonadism

**Fig. 3:**
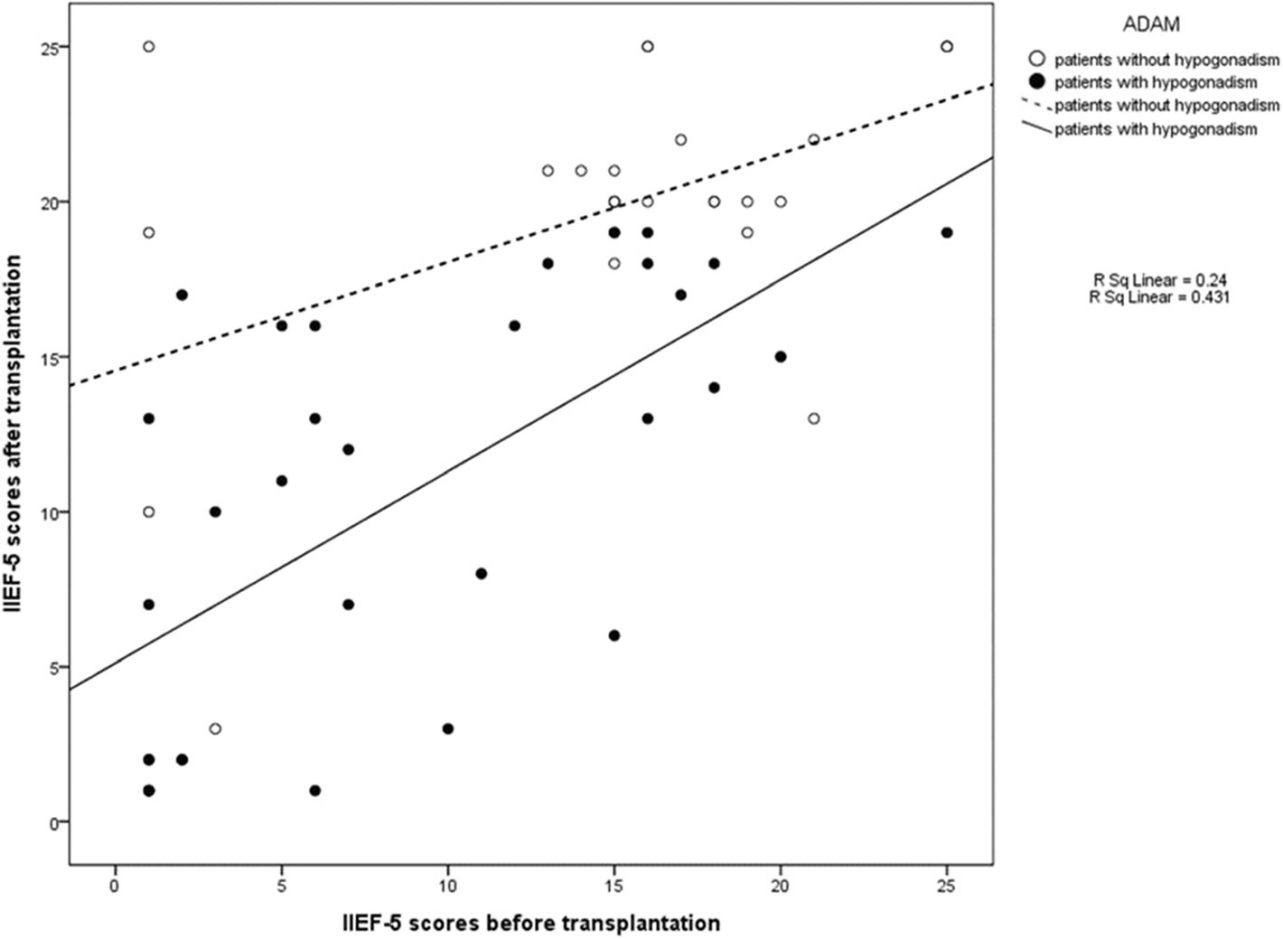
Correlation between erectile dysfunction before and after transplantation for patients with or without hypogonadism

We found that hypogonadism before LDLT was associated with improvement in erectile function after LDLT. There was a significant correlation between hypogonadism before LDLT and IIEF-5 score prior to LDLT (Fig. 3). In patients with ADAM questionnaire scores indicating no hypogonadism, the mean IIEF-5 score was 15.35 ± 6.89 prior to LDLT and 19.92 ± 4.92 after LDLT (*p* <0.01) (Table 3). In patients with ADAM scores indicative of hypogonadism, the mean IIEF-5 score was 8.75 ± 7.07 before surgery and 10.53 ± 6.65 after LDLT (*p* = 0.107).

**Table 3:** IIEF-5 scores before and after transplantation for each group

	IIEF-5 score		
	Before transplantation (mean ± SD)	After transplantation (mean ± SD)	*p* value
With hypogonadism	8.75 ± 7.07	10.53 ± 6.65	0.109
Without hypogonadism	15.35 ± 6.89	19.92 ± 4.92	0.001
HCV positive	9.24 ± 6.98	11.06 ± 7.81	0.247
HCV negative	12.73 ± 7.80	16.27 ± 6.97	0.001
With DM	10.48 ± 6.51	12.15 ± 7.40	0.094
Without DM	12.77 ± 8.53	17.00 ± 7.02	0.002

### Factors associated with post-LDLT erectile dysfunction

The existence of HCV infection was significantly associated with lower IIEF-5 scores (mean IIEF-5 score in patients with HCV = 11.06 ± 7.81 vs. mean IIEF-5 score in patients without HCV = 16.27 ± 6.97, *p* = 0.015). Patients with clinical evidence of DM had lower IIEF-5 scores than those without diabetes (IIEF-5 of patients with DM = 12.15 ± 7.40 vs. IIEF-5 of patients without DM = 17.00 ± 7.02, *p* = 0.013). Other parameters such as alcohol-related origin of liver disease, development of post-transplantation DM, and gender of donor were not associated with the development of post-LDLT erectile dysfunction or hypogonadism.

## Discussion

Determination of sexual health is challenging, especially in Asian men who are often reluctant to undergo evaluation of sexual function. The response rate among participants to questionnaires designed to survey sexual function has been reported to range from 22.5 % to 81 % [8, 11, 16]. In our study, however, the response rate was 85 %. The introductory letters written by the surgeon who performed the LDLT procedures most likely contributed to the relatively high compliance rate in our study. The etiology of sexual dysfunction is multi-factorial and ESLD is one of many factors associated with the disorder. Liver transplantation restores liver function and should, theoretically, result in improvements in sexual function [8]. However, immunosuppressive drugs, which are key to survival after liver transplantation, are known to impair sexual function [17]. Alterations in the hypothalamic-pituitary-gonadal axis, changes in estrogen-over-androgen ratio, and altered sex hormone transport have been shown to cause hypogonadism in patients with impaired liver function [16, 18, 19]. Decreased testosterone level, libido, testis size and even infertility have been reported in patients with cirrhosis [3, 20]. Foresta et al. reported decreased sex hormone binding protein levels and higher free testosterone in serum in patients after OLT, which may explain the improvement in physiological function after the operation, even under immunosuppressive therapy [4]. Park et al. reported that up to 60 % of men reported no improvement in erectile function after OLT [10]. Klein and colleagues reported improvement in sexual function including erectile function, sexual satisfaction and sexual desire and a statistical trend in improvement in IIEF scores after liver transplant [21]. In our study, we found that nearly half of the patients reported improvement in erectile function. Many factors contribute to ED, including drugs, neurogenic disorders, cavernosal disorders, hormones, psychological causes, surgery, aging, and diabetes [22]. In our study, higher IIEF-5 scores were associated with absence of hypogonadism after the operation. That is, improvement in erectile function after liver transplantation is mainly hormone related. Theoretically, irreversible damage of the hypothalamic-pituitary-gonadal axis by long-term consumption of alcohol implies the low possibility of improvement in sexual function after LDLT [23]. However, Huyghe E et al. reported no higher risk of erectile dysfunction in patients with end-stage liver disease secondary to alcohol consumption [8]. Burra et al. also reported that there was no significantly lower trend in IIEF score in patients with alcohol-induced ESLD [3]. Sorrell and Brown found that 40 % of patients developed erectile dysfunction and 25 % reported having a lower level of sexual satisfaction after liver transplantation [16]. Ho et al. reported that 32 % of patients developed de novo sexual dysfunction after OLT [24]. Cardiovascular disease, post-transplantation diabetes, alcohol abuse, antidepressants and angiotensin II receptor blockers have been shown to be associated with new onset erectile dysfunction after liver transplantation [8]. In our study, 12 % (n = 7) of our patients reported a decrease in erectile function after LDLT. The low incidence of de novo erectile dysfunction might be due to the relatively short follow-up period in our study.

There are several limitations in this study. First, immunosuppressive regimens after transplantation were not consistent throughout the study population. Therefore, we were not able to take pharmacologic factors into account. Second, our findings are limited by the relatively small sample size and short follow-up period. Third, we did not measure serum testosterone levels and therefore we do not have laboratory data to support the change in prevalence of hypogonadism. Nonetheless, to the best of our knowledge, this is the first study to compare changes in sexual function before and after LDLT. Large-scale studies with longer follow-up periods are needed to clarify the role liver transplantation plays in sexual function.

## Conclusions

LDLT results in improvement in erectile function. In addition, improvement in erectile function is associated with absence of hypogonadism before LDLT.
